# Genomic Insights into Abdominal and Intramuscular Fat Deposition in Chickens and Their Implications for Productivity Traits: A Systematic Review

**DOI:** 10.3390/ani16020260

**Published:** 2026-01-15

**Authors:** Olga Kochetova, Gulnaz Korytina, Yanina Timasheva, Irina Gilyazova, Askar Akhmetshin, Gulshat Abdeeva, Alexandra Karunas, Elza Khusnutdinova, Oleg Gusev

**Affiliations:** 1 Institute of Biochemistry and Genetics—Subdivision of the Ufa Federal Research Centre of the Russian Academy of Sciences (IBG UFRC RAS), 450054 Ufa, Russia; guly_kory@mail.ru (G.K.); gilyasova_irina@mail.ru (I.G.); akhmetshin.askar99@gmail.com (A.A.); gulshatik2001@mail.ru (G.A.); carunas@list.ru (A.K.); elzakh@mail.ru (E.K.); o.gusev.fo@juntendo.ac.jp (O.G.); 2 Life Improvement by Future Technologies (LIFT) Center, 121205 Moscow, Russia; 3 Department of Regulatory Transcriptomics for Medical Genetic Diagnostics, Juntendo University, Tokyo 113-8421, Japan

**Keywords:** fat accumulation in chickens, microRNAs, long non-coding RNAs, circular RNAs, genetic markers

## Abstract

Abdominal fat deposition in chickens significantly impacts production efficiency and is influenced by complex genetic and molecular mechanisms. This review summarizes current genomic and transcriptomic research on the regulation of adipogenesis and fat accumulation in chickens, highlighting key genes and loci identified through genome-wide association studies as well as other candidates involved in lipogenesis, lipolysis, and transcriptional regulation. Major metabolic pathways, including MAPK, AMPK, PI3K/AKT/mTOR, TGFβ1/Smad3, FoxO, JAK–STAT, Wnt/β-catenin, and Sonic Hedgehog signaling, are examined for their roles in fat deposition. The regulatory functions of non-coding RNAs, including microRNAs, long non-coding RNAs, and circular RNAs, are discussed, focusing on their interactions with target mRNAs and signaling networks that control lipid metabolism, adipocyte differentiation, and energy balance. Integrating insights from both avian and human studies, this review emphasizes the molecular mechanisms underlying adipogenesis and highlights potential strategies for genetic selection aimed at reducing excessive abdominal fat and improving poultry productivity.

## Simple Summary

Chicken production is strongly affected by the amount of fat that accumulates in the abdominal area, as excessive fat lowers efficiency and increases feed costs. This review explains what is currently known about the genetic and molecular factors that influence how chickens form and store fat. Research using large genetic databases has identified specific genes and regions of the genome that play important roles in the creation of new fat cells, the breakdown of stored fat, and the control of energy use in the body. This review also describes several major biological pathways that regulate these processes, explaining how they influence the balance between fat production and fat removal. In addition, it highlights the importance of non-coding molecules, such as small and long RNA fragments that do not form proteins but help control how genes work. These molecules shape many steps of fat development by interacting with target genes and with networks that regulate cell growth, metabolism, and energy balance. By bringing together findings from chicken research and relevant discoveries in humans, this review outlines promising molecular targets that could support breeding strategies aimed at reducing excessive abdominal fat and improving the overall efficiency and sustainability of poultry production.

## 1. Introduction

**Figure 1:**
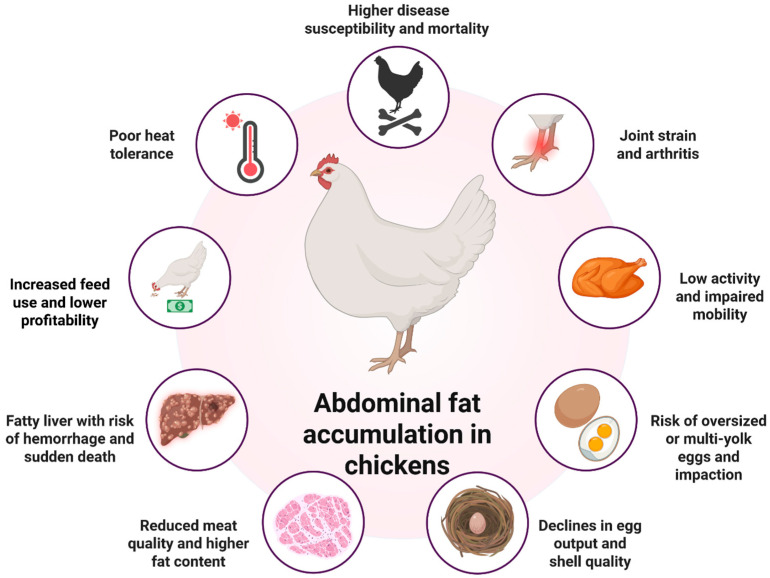
Adverse effects and risks associated with abdominal fat accumulation in chickens (Figure was created using the online tool “Scientific Image and Illustration Software BioRender”, https://biorender.com).

Obesity is a condition characterized by excessive fat accumulation in the body [1]. In poultry, it has become an increasing concern because it markedly worsens the overall health of birds and disrupts normal production parameters [2]. This disorder predominantly affects laying hens and broilers and is marked by disturbances in energy and lipid metabolism, excessive fat deposition, increased body mass, and reduced egg production [3]. In laying hens, excess adiposity is associated with substantial declines in egg output [4], erratic ovulation, ovarian dysfunction [5], the production of larger but fewer eggs [6], increased incidence of egg binding and prolapse [7], and reduced egg quality and hatchability [8]. These reproductive disturbances stem primarily from an energy imbalance that drives disproportionate fat deposition and disrupts normal reproductive physiology and egg formation [6] (Figure 1).

In broilers, selection for rapid muscle accretion, combined with hyperphagia, predisposes birds to adult obesity when feed is not restricted. Obese broilers exhibit elevated circulating insulin and leptin, alterations in lipid and lipoprotein metabolism comparable to human metabolic syndrome, and intensified systemic inflammation [5,9,10,11]. These birds also suffer reductions in economically important traits, including poorer feed efficiency [4], increased production costs [6], higher mortality [12], management difficulties [4], diminished fertility, and delayed sexual maturity [5]. As a result, both laying and broiler operations experience reduced efficiency and profitability (Figure 1).

Understanding the molecular mechanisms that regulate excessive fat accumulation is essential for designing effective preventive and corrective strategies, crucial for maintaining flock health, improving productivity, and enhancing economic sustainability. The pathological manifestations of obesity in chickens encompass structural, behavioral, metabolic, and reproductive disorders. Affected hens commonly display a less prominent keel bone and extensive abdominal fat deposits [13]. Reduced activity and impaired locomotion are frequent behavioral indicators. Production-related consequences include sharp declines in egg laying, compromised shell quality, oversized or multi-yolk eggs, and an elevated risk of egg binding, which can be fatal. Excessive infiltration of fat into the liver may lead to hepatic hemorrhagic syndrome, severely impairing hepatic function and causing sudden death due to internal bleeding. Heavy birds also undergo pronounced joint stress, often resulting in arthritis and chronic mobility issues. Their ability to dissipate heat is impaired, increasing susceptibility to heat stress. Furthermore, obesity compromises immune function, predisposing birds to infectious diseases and raising mortality rates and veterinary expenses. Higher feed intake is required to maintain their enlarged body mass, elevating production costs and diminishing profitability [14,15,16]. In broilers, excessive carcass fat reduces meat quality and market value [17].

Improper feeding practices, particularly overfeeding calorically dense diets, inadequate space for exercise, and prolonged confinement are among the common causes of obesity in chickens. Certain breeds, especially those selected for high body mass, exhibit a genetic predisposition to excessive weight gain [18]. Such birds may be more prone to overeating or more efficient at converting feed into fat, making them vulnerable to obesity even under moderate feeding regimes. Additionally, excessive abdominal fat correlates with elevated concentrations of saturated fatty acids and cholesterol in poultry meat, which can pose health concerns for humans, particularly with respect to cardiovascular disease risk [19].

Studying the similarities and differences between the human and chicken genomes provides valuable insights into evolutionary biology and comparative genomics [20]. Comparative analysis of these genomes makes it possible to identify genes unique to humans, genes unique to chickens, and genes shared by both species. Such information helps clarify how each species has evolved and adapted to its respective environment [21]. The chicken genome also offers significant potential for biomedical research. Chicks serve as important models for investigating human diseases, including cancer and infectious disorders. Moreover, understanding the genetic basis of chicken development and physiology can shed light on corresponding processes in humans [22].

The rapidly advancing field of poultry genomics has achieved substantial progress in unraveling the complex genetic architecture underlying fat deposition in chickens. Genome-wide association studies (GWAS) and transcriptomic analyses have identified numerous quantitative trait loci (QTL) and candidate genes involved in lipid metabolism, adipogenesis, and energy homeostasis [19].

The aim of this review is to provide a comprehensive and critical synthesis of genomic and transcriptomic studies addressing excessive abdominal fat deposition in chickens and its consequences for production traits. It also examines parallels between avian pathological phenotypes and human disorders stemming from impaired adipogenesis, with a particular focus on non-coding RNAs that modulate signaling pathways and regulate target mRNAs involved in lipid metabolism, adipogenesis, and energy homeostasis in both species.

## 2. Materials and Methods

The review was conducted in accordance with the *Animals* journal guidelines for review articles. Literature was evaluated with the objective of integrating current findings on the genetic and molecular mechanisms underlying abdominal fat accumulation in chickens, emphasizing its relevance to production performance and its broader biological parallels to human adipogenic dysfunction. Special attention was given to research on non-coding RNAs and their roles in key regulatory pathways governing lipid metabolism and adipogenic processes.

The literature search incorporated publications and datasets retrieved from the GWAS Atlas (https://atlas.ctglab.nl/, accessed on 6 November 2025), NCBI (https://www.ncbi.nlm.nih.gov, accessed on 6 November 2025), Ensembl (https://www.ensembl.org/index.html, accessed on 11 November 2025), the ChickenGTEx Atlas (https://ngdc.cncb.ac.cn/chickengtex, accessed on 20 November 2025). Studies published between 1993 and November 2025 were evaluated, and when findings differed across sources, the more recent evidence was prioritized for inclusion. The search strategy employed the keywords “chicken” AND (“obesity” OR “abdominal fat deposition” OR “intramuscular adipose deposition”) AND (“pathological traits” OR “productivity”) AND “gene” (“microRNA and lipogenesis” OR “long non-coding RNAs and lipogenesis” OR “circular RNAs and lipogenesis”). Articles that included three keywords simultaneously and free full text articles in NCBI were analyzed. The description is presented in Supplementary Figure S1, which outlines the principles of compliance with PRISMA guidelines. Records removed for other reasons included articles related to microbiome research, as well as articles unrelated to genetic research. Articles published only in the Russian language were analyzed in the Google Scholar database (https://scholar.google.com, accessed on 25 November 2025) The study included six articles in Russian. Articles identified through the database search were independently screened by two PhD-level researchers in biological sciences. Inclusion criteria required that sources be peer-reviewed articles written in English or Russian. Studies were excluded if they involved species other than chickens or consisted solely of abstracts, conference proceedings, or unpublished data. Searches focusing on non-coding ribonucleic acids (RNAs) utilized specialized databases, including RNAcentral (https://www.rnacentral.org, accessed on 25 November 2025), miRDB (https://mirdb.org, accessed on 25 November 2025), and LNCipedia (https://lncipedia.org, accessed on 25 November 2025), to identify relevant sequences, functional annotations, and experimentally validated targets.

Databases GWAS Atlas (https://atlas.ctglab.nl/, accessed on 6 November 2025), Ensembl (https://www.ensembl.org, accessed on 11 November 2025), the ChickenGTEx Atlas (https://ngdc.cncb.ac.cn/chickengtex, accessed on 20 November 2025) and GeneCards (https://www.genecards.org, accessed on 21 November 2025) were used to create tables in Supplementary Materials.

Annotation and meta-analysis of GWAS data were performed using RefSeq annotations for the Gallus_gallus-5.0 assembly (GCF_000002315.4). Data processing and visualization were carried out with custom Python 3.11.6 scripts employing the Pandas 2.2.2, NumPy 1.26.4, Matplotlib 3.9.1, Seaborn 0.13.2, and adjustText 1.1.1 libraries (https://www.ncbi.nlm.nih.gov/datasets/genome/GCF_000002315.4 (accessed on 4 December 2025) and Supplementary File S1: Script for data processing and visualization).

The review article was evaluated and approved by the local Ethical Committee. A positive conclusion was received: protocol No. 19 (4 December 2025).

## 3. Key Genes and Signaling Pathways Involved in Fat Deposition in Chickens

### 3.1. Genes and Loci Identified Through GWAS

Efforts to identify major genetic markers associated with abdominal fat and growth traits in chickens have employed GWAS, FST, and π-analysis [23]. Body mass is a complex quantitative trait shaped by both genetic influences and environmental conditions [24]. GWAS have been widely applied to elucidate the genetic architecture of growth-related traits across various species, and a curated GWAS database including chicken analyses is now available (GWAS Atlas). Loci associated with weight-gain traits in chickens are summarized in Supplementary Table S1.

Several genes identified through GWAS, including *ACTA1*, *IGF2BP1*, *TAPT1*, *LDB2*, *PRKCA*, *TGFBR2*, *GLI3*, *SLC16A7*, *INHBA*, *BAMBI*, *APCDD1*, *GPR39*, and *GATA4*, have been highlighted as key candidates influencing rapid growth in chickens [25]. Genes identified through GWAS as key candidates influencing rapid growth in chickens include *ACTA1*, *IGF2BP1*, *TAPT1*, *LDB2*, *PRKCA*, *TGFBR2*, *GLI3*, *SLC16A7*, *INHBA*, *BAMBI*, *APCDD1*, *GPR39*, and *GATA4* [25]. Additional genes associated with body-mass traits include *CAPZA2*, *MET*, *MYCBP2*, *ENSGALG00000029931*, *ENSGALG00000048550*, *ENSGALG00000038613*, *NUFIP1*, *GPALPP1*, *gga-mir-12214*, *SUCLA2*, *NUDT15*, *MED4*, *ENSGALG00000047974*, *ITM2B*, *CAB39L*, *SETDB2*, *KPNA3*, *ENSGALG00000053103*, *TRIM13*, *KCNRG*, *ENSGALG00000048894*, *ENSGALG00000017031*, *SLC25A15*, *ENSGALG00000054550*, *ENSGALG00000051912*, *PLEKHA8*, *COLQ*, *ENSGALG00000049188*, *CNOT6L*, *ENSGALG00000051573*, *U6*, *gga-mir-1697*, and *TOX2* [26].

#### Annotation and Analysis of GWAS Metadata for Economically Valuable Traits in Chickens

A multivariate GWAS meta-analysis revealed that BW-associated variants display a consistent, ordered distribution across three developmental stages [27]. Loci on chromosomes 1 and 4 exert primary regulatory influence during the early (1–7 weeks) and intermediate (8–22 weeks) growth phases. Genes on chromosome 1 predominantly affect early growth, while genes on chromosome 4 exert a stronger influence during the intermediate phase. This temporal specificity is reflected in the distribution of variants associated with body weight (BW) measured at different growth stages (BW56, BW70, BW84), indicating distinct regulatory roles of these chromosomes throughout chicken development.

**Figure 2:**
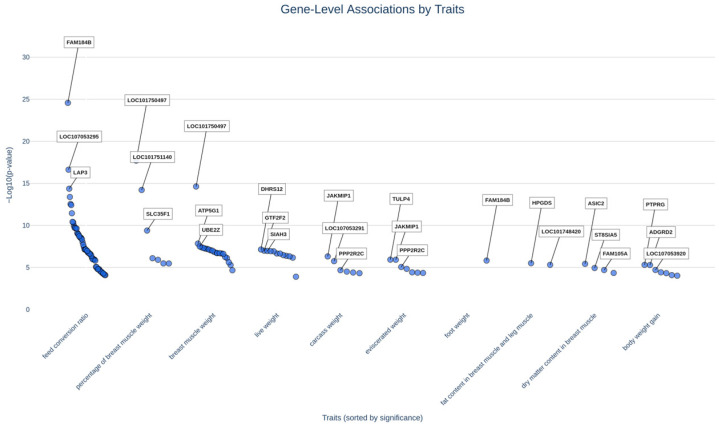
Mega–ridge plot showing associations between weight-gain traits in chickens and gene polymorphisms (*Y*-axis represents the median −log10 *p*-value across all loci within each gene).

Subsequently, we analyzed GWAS metadata for economically important poultry traits. Figure 2 summarizes gene–trait associations related to weight-gain characteristics in chickens. The strongest meta-analytic association was identified for *FAM184B* (family with sequence similarity 184, member B, transcript variant X1) on chromosome 3 (chr3:62914374, *p* = 9.77 × 10^−42^), linked to feed conversion ratio [28]. A significant association with feed conversion ratio was also observed for *LAP3* on chromosome 4 (chr4:76541002, *p* = 6.51 × 10^−21^). Leucine aminopeptidase 3 catalyzes the hydrolysis of terminal leucine residues from peptide substrates and participates in cell proliferation and myogenic differentiation [29]. *LOC101750497* showed strong associations with both percentage of breast muscle weight and breast muscle weight (chr3:64294177, *p* = 1.98 × 10^−18^ and *p* = 2.37 × 10^−15^), while the uncharacterized locus *LOC107053295* (transcript variant X1) was associated with feed conversion ratio (chr4:77869526, *p* = 1.98 × 10^−17^) (Supplementary Table S1).

**Figure 3:**
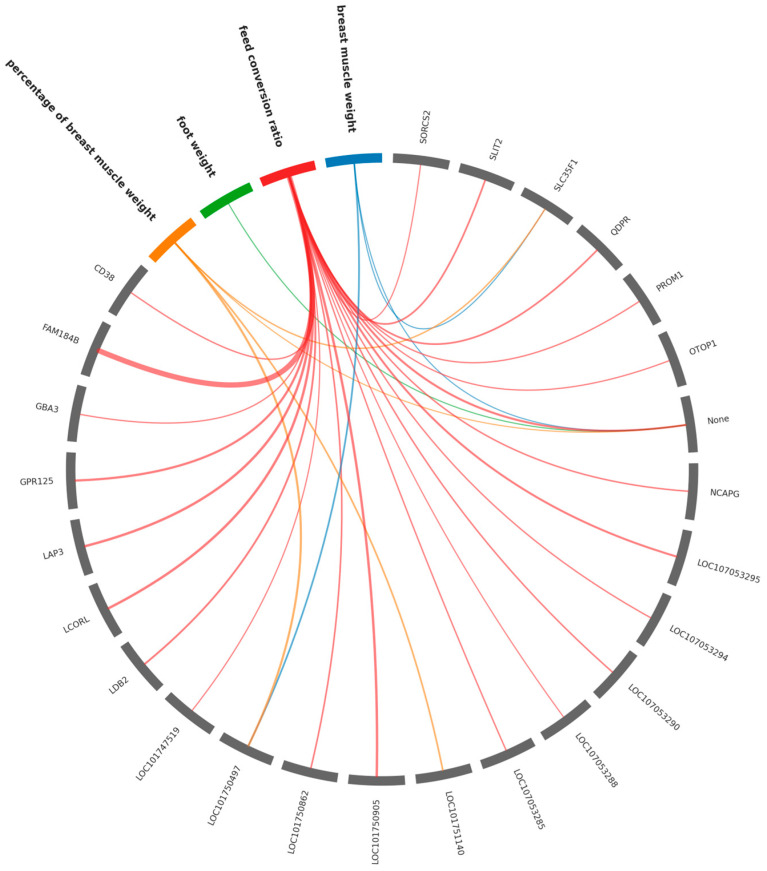
Chord diagram illustrating associations between polymorphic loci/genes and quantitative meat productivity traits in chickens. Colored sectors and bold labels represent different phenotypic traits, gray sectors with normal font labels represent genes. The links connect significant gene-trait pairs, with the link color corresponding to the associated trait. The thickness of the connecting lines is proportional to the significance of the association (−log10 *p*-value).

Figure 3 illustrates gene-trait relationships for key meat productivity traits (feed conversion ratio, percentage of breast muscle weight, breast muscle weight) using a chord diagram. Supplementary Table S2 lists 108 genes associated with 10 production traits, including 29 genes with the strongest significance across six economically relevant characteristics.

### 3.2. Other Genes Potentially Involved in Abdominal Fat Deposition

Most studies on this topic focus on genes that govern lipid metabolism in chickens, although their direct involvement in abdominal fat accumulation has not been firmly established [24]. Fat deposition in chickens results from coordinated lipogenesis, lipid transport, and the activity of genes responsible for lipid storage (*PPARG*, *FASN*, *SCD*, *FABPs*, *LPL*, *PLINs*). These genes act through PPAR signaling (peroxisome proliferator-activated receptors), insulin signaling, PI3K/AKT signaling, AMPK/mTOR and MAPK pathways, as well as steroid biosynthesis routes, and are linked to tissue-specific gene expression and emerging regulatory targets [30].

Adipogenesis and fat accumulation in chickens are controlled by a wide array of signaling cascades involving enzymes, transport proteins, transcription factors, regulatory non-coding RNAs, and mitochondrial genes that govern lipid synthesis, uptake, and storage in tissues. Key genes repeatedly identified in transcriptomic and co-expression studies include canonical lipogenic and lipid-transport genes, along with coactivators and regulators of mitochondrial function [30].

#### 3.2.1. Lipogenesis Genes

Several key genes constitute the core of the lipogenesis pathway, which governs the synthesis of fatty acids and triglycerides in the adipose tissue of chickens and plays a decisive role in abdominal fat accumulation. A substantial majority of the fatty acids stored in chicken adipose tissue originate from hepatic lipogenesis, which proceeds far more vigorously in the liver than in adipocytes [31] (Table 1).

Central enzymes in this pathway, including fatty acid synthase (FASN), acetyl-CoA carboxylase alpha (ACACA/ACCα/β), stearoyl-CoA desaturase (SCD), and stearoyl-CoA desaturase 5 (SCD5), catalyze de novo fatty acid synthesis and desaturation. Numerous studies have documented heightened activity of these enzymes in the adipose depots of chickens [32,33,34,35].

Among these, FASN occupies a central position. It directs the de novo synthesis of fatty acids in the liver and exerts a defining influence over overall lipogenic capacity. Its expression is substantially elevated in birds that exhibit pronounced abdominal fat deposition [36]. FASN activity is orchestrated by several transcriptional regulators, foremost among them sterol regulatory element binding protein 1 (SREBP-1), which induces lipogenesis gene expression in response to insulin and dietary signals [37]. Peroxisome proliferator-activated receptor gamma (PPAR-γ), a principal regulator of adipocyte differentiation, also augments lipogenesis gene transcription and promotes adipogenesis [38]. Activation of the PPAR-γ pathway has been associated with increased adiposity and intensified accumulation of abdominal fat in broilers [34]. Although FASN, ACC, PPAR-γ, and related genes are dominant contributors to lipid accretion, comparative analyses reveal species-specific patterns in their transcriptional profiles [38]. Additional regulatory elements, including PPARG, PPARGC1A (PGC-1α), members of the retinoid X receptor family, and INSIG1, serve as integrative nodes coordinating lipogenesis pathways in chicken tissues [32,35].

Genes implicated in lipid uptake, transport, and intracellular fatty acid trafficking include *CD36*, *LPL*, *APOA1*, *APOB*, *APOV1*, and several fatty acid-binding proteins (*FABP3*, *FABP4*, *FABP5*, *FABP7*). These genes are consistently reported to be overexpressed in chicken adipose tissues [32,33,34]. *FABP4* is expressed most abundantly in abdominal fat, where it plays a prominent role in fatty acid handling and metabolic turnover [39]. The protein coded by the *FABP4* gene is thought to influence lipid deposition through mechanisms that include lipolysis, facilitating the release and subsequent utilization of fatty acids as an energy source [40]. Several FABP genes participate in the PPAR-regulated signaling axis that governs lipid deposition; in one study, *FABP3*, *FABP4*, and *FABP7* were downregulated, whereas *FABP5* expression increased [34]. Another family member, *FABP2*, contributes to the control of lipogenesis, and an epistatic interaction between *FABP2* and *ACACA* has been associated with differences in abdominal fat weight and proportion [41].

*GPAM* gene encodes mitochondrial glycerol-3-phosphate acyltransferase, an enzyme that catalyzes the first committed step of triglyceride synthesis by esterifying glycerol-3-phosphate. This reaction is indispensable for energy storage and fat accumulation in chickens [1]. Its expression is strongly modulated by dietary factors, particularly by the fatty acid composition of the diet. When chickens are fed diets enriched with unsaturated fatty acids, hepatic *GPAM* expression rises, correlating with increased triglyceride synthesis and greater fat deposition [42].

*GPAM* is also a direct target of miR-223, a micro ribonucleic acid (miRNA) involved in adipocyte differentiation; levels of miR-223 decline in the breast muscle during the later phases of the laying cycle and show a negative correlation with *GPAM* expression [31]. Functional enrichment analyses indicate that *GPAM* contributes to fatty acid homeostasis, triglyceride biosynthesis, and wider aspects of lipid metabolism [43]. Further evidence identifies *GPAM* as a gene linked to lipid-metabolic signaling, with its upregulation promoting triglyceride synthesis, a core step in fat storage [44].

Genes from the ELOVL fatty acid elongase family, particularly *ELOVL6*, extend long-chain fatty acids and thereby influence abdominal fat deposition in chickens [45]. Variation in the expression of *ELOVL1*, *ELOVL2*, *ELOVL5*, and *ELOVL6* has been linked to breed-specific differences in lipid accumulation [46]. Genetic variation within these loci shapes distinct phenotypes, including diverse patterns of fat deposition [31,47]. *ELOVL6* also interacts with dietary elements such as fatty acid composition, illustrating the role of gene-environment interactions in determining lipid metabolism and fat accretion in chickens [48].

ELOVL5 catalyzes the elongation of docosapentaenoic acid to tetracosapentaenoic acid and contributes to the formation of docosahexaenoic acid from alpha-linolenic acid, a pathway more active in chickens than in many other species [49]. Its expression in adipose tissue depends on the dietary intake of alpha-linolenic acid and supports docosahexaenoic acid accumulation and associated changes in lipid metabolism [50]. The product of *ELOVL7* participates in the regulation of β-oxidation and adipogenesis, processes closely linked to fat accretion [50].Together, these lipogenesis genes and their regulators illustrate the intricacy of adipogenic mechanisms that govern abdominal fat deposition in chickens.

#### 3.2.2. Lipolysis Genes

Lipolysis is a catabolic pathway in which stored triacylglycerols are hydrolyzed to free fatty acids and glycerol, supplying metabolic energy derived from endogenous lipid reserves [51]. The enzymes involved in this process exert substantial influence over adipose tissue dynamics, as they regulate the release and utilization of fatty acids from lipid stores [51] (Table 1).

Among the central genes governing lipolytic activity is lipoprotein lipase (*LPL*), the enzyme responsible for hydrolyzing triacylglycerols contained in circulating lipoproteins to produce free fatty acids. This reaction is fundamental for the uptake of fatty acids by adipose tissue and skeletal muscle [1]. Increased *LPL* expression is associated with enhanced lipolytic capacity and reduced abdominal fat deposition, highlighting its key role in avian lipid homeostasis [52]. Notably, the regulation of *LPL* in chickens diverges from that observed in mammals, as neither fasting nor overfeeding alters *LPL* activity or transcript abundance in avian adipose tissue, suggesting relatively low nutritional sensitivity of this gene in birds [53].

Another major component of the lipolytic network is carnitine palmitoyltransferase 1 (CPT1), the enzyme that facilitates the mitochondrial import and subsequent β-oxidation of long-chain fatty acids [54]. This pathway is indispensable for the conversion of fatty acids into usable energy and for preventing excessive lipid accumulation. The CPT1A and CPT1B isoforms hold particular relevance in chickens, as they govern lipid flux in tissues characterized by substantial fat deposition, such as abdominal and intramuscular fat [1]. *CPT1A* expression is elevated in the liver and various adipose depots, indicating broad involvement in lipid turnover [55]. During adipocyte differentiation, increases in lipid droplet formation parallel rising *CPT1A* expression, whereas inhibition of *CPT1A* leads to greater lipid accretion. CPT1A does not act as an isolated rate-limiting factor; rather, it operates in coordination with additional transport proteins such as CROT, which transfers acylcarnitines from peroxisomes to mitochondria [56]. Its activity is further modulated by several signaling cascades. For instance, CPT1A promotes adipocyte proliferation and restrains lipid accumulation through MAPK-dependent mechanisms [56]. Elevated *CPT1* expression in skeletal muscle and subcutaneous adipose tissue also suggests a role in shaping intramuscular fat content, potentially via regulation by PPARA [57].

A further enzyme central to fatty-acid catabolism is acyl-CoA dehydrogenase, long-chain (ACADL), which initiates the mitochondrial β-oxidation of long-chain fatty acids [58]. Increased *ACADL* expression during embryonic development has been linked to intramuscular fat accumulation in broilers, underscoring the importance of oxidative lipid pathways in determining abdominal fat deposition [59]. Monoglyceride lipase (MGLL) also contributes significantly to lipolysis by hydrolyzing monoglycerides into free fatty acids and glycerol, promoting the mobilization of stored lipids [60]. The *APP* gene encodes the amyloid beta precursor protein, a transmembrane receptor that undergoes proteolytic processing to generate several bioactive peptides [1]. APP enhances transcription through interaction with APBB1/KAT5 and suppresses Notch signaling [1]. Its expression declines during lipolysis [61]. Acetyl-CoA acetyltransferase 1 (ACAT1), which participates in ketone-body metabolism and terpenoid biosynthesis, is similarly downregulated during lipolysis [62].

#### 3.2.3. Transcription Factors

Transcription factors play a central role in regulating the expression of genes involved in adipogenesis in chickens. By binding to specific DNA motifs within promoter regions, they orchestrate the transcription of genes that control metabolic pathways governing fat accumulation. Understanding the functions of these regulatory proteins provides critical insight into the genetic mechanisms underlying abdominal fat deposition in chickens [63] (Table 1).

Peroxisome proliferator-activated receptor gamma (PPARγ) is among the most influential transcription factors associated with abdominal fat deposition. This ligand-activated regulator is essential for adipocyte differentiation, lipid storage, and overall lipid homeostasis [64,65]. Its activity spans key steps in adipogenesis, including the release of fatty acids from triglycerides, intracellular fatty-acid trafficking, and fatty-acid esterification, mediated through transcriptional activation of genes such as *FABP4* and *LPL* [66]. PPARγ cooperates with CEBPA, a pivotal regulator of adipocyte maturation, to stimulate the expression of molecular factors that promote lipid accumulation [36]. In chickens, enhanced PPARγ activity is closely associated with increased abdominal fat deposition [67]. Hepatic PPARγ expression strongly correlates with abdominal fat content, as lipogenesis in birds occurs primarily in the liver, which is markedly more lipogenic than adipose tissue [68].

CEBPA itself regulates genes involved in lipid metabolism and glucose homeostasis, acting synergistically with PPARγ to amplify the expression of lipogenesis genes [31,69]. In contrast, C/EBPZ functions as a transcriptional repressor, modulating key adipogenic regulators including PPARγ, FASN, and LPL. While C/EBPα and C/EBPβ facilitate adipocyte differentiation, C/EBPZ inhibits this process by suppressing essential transcriptional programs. Overexpression of C/EBPZ reduces RNA polymerase II recruitment in preadipocytes, highlighting its broad inhibitory role. Its activity enhances GATA2 and FABP4 expression while suppressing PPARγ, C/EBPα, FASN, and LPL transcription, thereby slowing adipocyte maturation [63,70].

Sterol regulatory element-binding protein 1 (SREBP1) is a key regulator of lipid metabolism, directing the expression of genes involved in fatty-acid and cholesterol synthesis. Its activation is driven by insulin and nutritional signals, emphasizing its role in shaping abdominal fat accumulation in chickens [37,71]. Insulin-induced gene 1 (*INSIG1*) is closely linked to this axis, suppressing SREBP1 activation and modulating the expression of lipogenesis enzymes [37]. FOXO1 contributes to adipocyte biology by inhibiting PPARγ transcriptional activity, thereby limiting adipogenesis [37]. Reduced *FOXO1* expression in adipose tissue enhances thermogenesis processes, increasing energy expenditure and supporting weight reduction [72]. Its expression is regulated by insulin, while *FOXO1* itself controls genes involved in lipolysis and fatty-acid oxidation [73]. Thyroid hormone responsive spot 14 (*THRSP*) is also associated with abdominal fat deposition, further highlighting the complexity of transcriptional regulation [74].

Collectively, these findings underscore the broad and interconnected roles of transcription factors in regulating both lipogenesis and lipolysis.

Network analyses have identified additional hub genes within modules linked to energy metabolism and lipid deposition, including mitochondrial and energy-sensing components such as *PRKAG2*, *ATP5B*, *NDUFAB1*, and *NDUFA9* [35].

Reduced expression of lipolytic genes limits fat breakdown, favoring anabolic activity and promoting fat accumulation, particularly in abdominal adipose tissue. Conversely, heightened expression of lipogenesis genes reflects increased metabolic activity and greater fat deposition [75]. A more comprehensive understanding of these regulatory networks will illuminate the mechanisms underlying abdominal fat accumulation in chickens and support breeding strategies aimed at reducing excessive adiposity.

### 3.3. Metabolic Pathways Regulating Fat Deposition in Chickens

#### 3.3.1. MAPK Signaling Pathway

**Figure 4:**
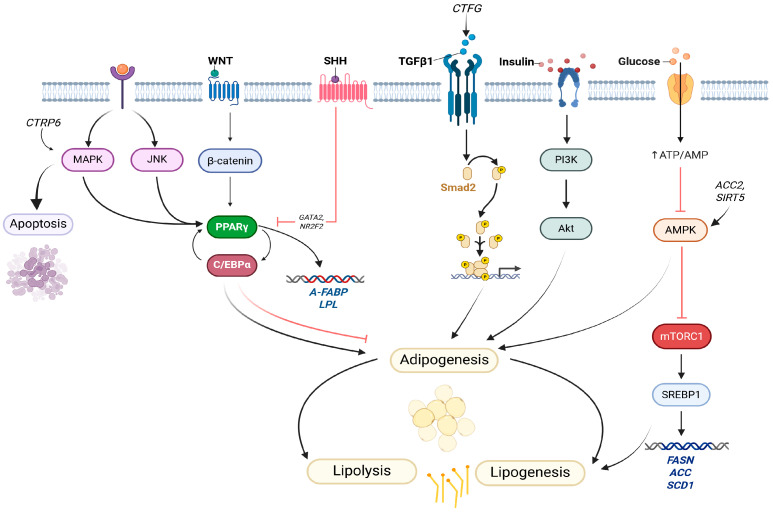
Key metabolic pathways governing adipogenesis, lipogenesis, and lipolysis in chickens (Figure was created using the online tool “Scientific Image and Illustration Software BioRender”, https://biorender.com).

The analysis of gene enrichment associated with excessive fat deposition in chickens has identified more than thirty-nine signaling pathways, among which the MAPK, mTOR, and FoxO cascades appear particularly influential [76]. Several studies indicate that the MAPK pathway modulates lipid metabolism and adipogenesis partly through its interaction with the PPAR signaling network [77,78,79]. Inhibition of MAPK/JNK signaling has been shown to markedly reduce the formation of lipid droplets, underscoring its importance in regulating adipocyte differentiation in chickens [80] (Figure 4).

Further evidence suggests that, in foam cells, MAPK/JNK signaling can phosphorylate PPARγ, thereby altering its transcriptional activity and influencing adipogenesis [81]. The adipokine C1q/tumor necrosis factor-related protein 6 (CTRP6) has also been implicated in the proliferation and differentiation of intramuscular and subcutaneous adipocytes through MAPK-dependent mechanisms [82]. Excessive accumulation of reactive oxygen species is known to activate MAPK signaling, which can trigger apoptosis, metabolic dysregulation, and inflammatory responses through cross-talk with the NF-κB cascade [83,84] (Figure 4).

#### 3.3.2. AMPK and PI3K/AKT/mTOR Signaling Pathways

Co-expression network analysis indicates that genes involved in AMPK signaling (AMP-activated protein kinase) and components of the mTOR pathway (the mammalian target of rapamycin) participate in both fatty acid oxidation and fatty acid synthesis [85]. AMPK represents a central regulatory pathway that governs fatty acid biosynthesis, the metabolism of unsaturated fatty acids and cholesterol, and multiple stages of adipogenesis by coordinating diverse biological processes [75,86]. AMPK, mTOR, and insulin/PI3K–AKT signaling jointly modulate energy homeostasis and nutrient utilization; the gene products associated with these cascades control lipogenesis and pyruvate metabolism [35,85] (Figure 4).

In mammals, AMPK activity shows a negative correlation with fat accumulation but a positive association with muscle development [87]. Activation of AMPK enhances fatty acid oxidation through phosphorylation of acetyl-CoA carboxylase 2 (ACC2) [88]. It additionally influences lipid aggregation within myocytes by modulating mRNA m6A demethylation via FTO [89]. By suppressing adipogenic markers such as PPARG, C/EBPα, and FABP4, AMPK acts as an inhibitor of adipocyte differentiation [90]. Activation of AMPK by sirtuin 5 (SIRT5) has been shown to inhibit the MAPK signaling cascade, thereby limiting preadipocyte differentiation and lipid deposition in bovine cells [91].

Insulin- and adipokine-mediated signaling contributes to nutrient processing within muscle tissue and subsequently to lipogenesis, while maintaining the downstream effects of PI3K/AKT activation [35,85,92] (Figure 4). The cellular response to insulin regulates blood glucose levels by promoting glucose uptake in muscle and adipose tissue, thus facilitating energy storage in fat depots, the liver, and muscle through stimulation of lipogenesis, glycogen synthesis, and protein synthesis [93]. The insulin pathway is therefore a fundamental biochemical system governing glucose and lipid metabolism, protein synthesis, cellular growth, differentiation, survival, and apoptosis [94,95].

The PI3K/AKT pathway orchestrates numerous physiological processes by activating key downstream effectors that regulate the cell cycle, growth, and proliferation [96] (Figure 4). The IGF/PI3K/AKT axis has been implicated in controlling the transcriptional activity of MyoD family genes and has been shown to stimulate C2C12 myoblast differentiation [97]. Evidence also suggests a functional interaction between glucose and insulin in regulating myogenesis, as insulin influences both PI3K/AKT signaling and glucose transport via translocation of the facilitated glucose transporter GLUT4 [98].

#### 3.3.3. TGFβ1/Smad3, FoxO, and JAK–STAT Signaling Pathways

Components of the TGFβ1/Smad3, FoxO, and JAK–STAT signaling pathways participate in cell-cycle regulation, cellular stress responses, and cross-talk among intracellular cascades during adipogenesis [99]. Fat deposition largely results from the differentiation of preadipocytes, whereas excessive adiposity develops when differentiated preadipocytes accumulate beyond normal levels [63].

The importance of the TGFβ1/Smad3 pathway in adipocyte development has been demonstrated (Figure 4), with evidence that inhibition of adipocyte maturation occurs through the action of connective tissue growth factor (CTGF) on this signaling cascade. Expression of CTGF in abdominal adipose tissue is markedly higher in lean chickens than in those with elevated fat deposition [100]. Studies in chicken models of obesity further show that TGFβ1 does not alter the transcription of appetite-regulating neuropeptides. Nonetheless, administration of TGFβ1 induces Smad2 phosphorylation in the hypothalamus and suppresses feed intake without changing the expression of hypothalamic neuropeptides involved in appetite control, including neuropeptide Y (NPY), proopiomelanocortin (POMC), agouti-related peptide (AgRP), and corticotropin-releasing hormone (CRH) [101].

#### 3.3.4. Wnt/β-Catenin Signaling Pathway

The Wnt pathway is a highly conserved and evolutionarily ancient regulatory system that governs tissue development and homeostasis by controlling cellular proliferation, differentiation, migration, and apoptosis (Figure 4). Its activity depends on β-catenin and is commonly referred to as the Wnt/β-catenin signaling pathway [102]. This pathway functions as a molecular switch that balances myogenesis and adipogenesis. Activation of Wnt signaling promotes myogenic differentiation, whereas adipogenic differentiation is suppressed through the inhibition of PPARG and C/EBPα expression [103]. Enhanced Wnt signaling reduces fat formation by downregulating these adipogenic genes, while inhibition of β-catenin has the opposite effect, facilitating PPARG expression and promoting adipocyte differentiation [104].

#### 3.3.5. Sonic Hedgehog (SHH) Signaling Pathway

An expanding body of research highlights the influence of the Sonic hedgehog (SHH) signaling cascade on adipogenesis and lipogenesis. Activation of this pathway has been shown to suppress adipocyte formation in mouse embryonic fibroblast cell lines [105]. It has been proposed that a reduction in SHH signaling is necessary, though not sufficient on its own, to initiate adipocyte differentiation [106] (Figure 4).

SHH signaling is thought to direct stem cells toward myogenic or osteogenic lineages, whereas suppression of this pathway enhances adipogenic differentiation [102]. Further evidence demonstrates that SHH signaling counteracts adipogenesis by inducing the expression of GATA2, a transcription factor that interacts directly with C/EBPα and PPARγ, thereby blocking the pro-adipogenic activity of PPARγ [107]. In addition, SHH signaling negatively regulates adipogenesis through the transcription factor NR2F2, which binds to the promoter regions of C/EBPα and PPARG and represses their expression [107].

#### 3.3.6. Cross-Talk Between MAPK and PI3K/AKT/mTOR Pathways in Avian Lipogenesis

In chickens, the MAPK and PI3K/AKT/mTOR signaling pathways function as the principal signal transduction routes that integrate hormonal signals, such as insulin and adiponectin, with nutritional cues to regulate lipid homeostasis. Their interaction forms a complex regulatory network that promotes fat synthesis (lipogenesis) while generally suppressing lipolysis. The PI3K/AKT/mTOR axis represents the dominant signaling pathway controlling hepatic lipid synthesis in chickens, with the liver serving as the primary site of de novo lipogenesis in birds [108]. mTORC1 activates sterol regulatory element-binding protein 1 (SREBP1), a key transcriptional regulator of lipogenic genes [109]. Enzymatic regulation is further enhanced through increased activity of major lipogenic enzymes, including fatty acid synthase (FASN) and acetyl-CoA carboxylase (ACC), resulting in elevated lipid accumulation [110]. In parallel, activation of the MAPK/ERK pathway often acts synergistically with PI3K signaling to enhance the expression of PPARγ, a transcription factor essential for adipocyte differentiation and lipid storage [111].

## 4. The Role of Non-Coding RNAs in the Regulation of Adipogenesis

Advances in epigenetic research have revealed that non-coding ribonucleic acid (ncRNAs), including micro ribonucleic acid (miRNAs), long non-coding ribonucleic acid (lncRNAs), and circular ribonucleic acid (circRNAs), play diverse regulatory roles in the formation of abdominal fat in chickens [112,113]. According to the miRBase database (https://mirdb.org, accessed on 25 November 2025), Gallus gallus possesses 882 microRNA precursors that give rise to 1232 mature microRNAs, many of which are conserved across species, implying that they may perform comparable functions [46].

Epigenetically mediated metabolic reprogramming is essential before hatching, enabling the chick to transition from reliance on stored yolk lipids to the utilization of carbohydrate-based feed [114]. It has been shown that the expression of more than 800 mRNAs and 30 microRNAs changes in the embryonic liver from day 18 of development to day 3 post-hatch, and many of these differentially expressed transcripts are associated with metabolic processes [46].

### 4.1. MicroRNA-Mediated Regulation of Adipogenesis

Hepatic lipid metabolism is closely connected to overall fat deposition, and understanding the molecular regulation of lipogenesis and fatty acid synthesis can shed light on the mechanisms underlying obesity and excessive fat accumulation in chickens [31,115]. A wide variety of distinct microRNAs has been shown to participate in the processes underlying intramuscular fat deposition in chickens [116]. Table 2 summarizes the most influential miRNAs implicated in the regulation of adipogenesis in this species.

**Figure 5:**
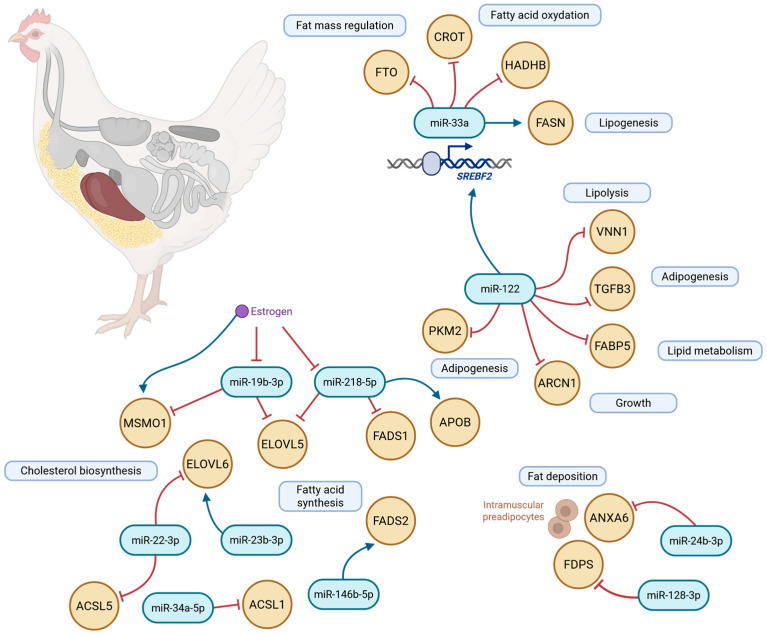
Key microRNA and their target genes in metabolic processes underlying chicken adipogenesis (Figure was created using the online tool “Scientific Image and Illustration Software BioRender”, https://biorender.com).

The miR-33 family participates in lipid metabolic control and encompasses two key miRNAs (miR-33a and miR-33b) encoded within intronic regions of the *SREBF2* and *SREBF1* genes, respectively [118]. MiR-33a is highly conserved across species, whereas miR-33b is predominantly expressed in large mammals and is absent in chickens. The intron 16 of *SREBF2* encodes miR-33, which modulates the expression of numerous genes involved in cholesterol, fatty acid, triglyceride, and phospholipid synthesis and uptake [71] (Figure 5). MiR-33 plays a central role in lipid metabolism and energy homeostasis by suppressing expression of the Fat mass and obesity-associated (*FTO*) gene in chicken hepatocytes. Its hepatic expression increases from day 0 to day 49 of development, while FTO expression decreases throughout this period, indicating negative regulation of *FTO* by miR-33 [132]. Additional research has shown that miR-33 affects fatty acid oxidation in the liver by repressing the genes *CROT* and *HADHB*, which encode enzymes essential for β-oxidation [71]. It has also been linked to regulation of the key lipogenic gene *FASN* [120].

MiR-122 is one of the most abundant hepatic miRNAs [119]. Strongly conserved across vertebrates, it contributes to cholesterol synthesis and lipid metabolism [120] and is integral to hepatic growth, development, lipogenesis, and liver pathology [128,133] (Figure 5). The *VNN1* gene, which encodes Vanin-1, contains a miR-122 binding site in its 3′-UTR, and its expression is suppressed by miR-122 in chicken hepatocytes, underscoring the miRNA’s role in lipid metabolism and hepatic energy homeostasis [133]. Additional targets in chickens include *TGFB3*, *FABP5*, *ARCN1*, *PKM2*, *FASN*, *SCD*, *ACACA*, *P4HA1*, *SREBF1*, and *SREBF2*, all of which are central to lipid metabolic pathways [133,134] (Figure 5).

MiR-19b-3p, a highly conserved miRNA across species, plays a dual role in lipid metabolism by promoting triglyceride synthesis while suppressing intracellular cholesterol formation through direct regulation of *MSMO1* and *ELOVL5* (Figure 5). Its expression is further modulated by estrogen at the post-transcriptional level [126]. Similarly, miR-5 influences adipogenesis by targeting the 3′-UTR of C/REB1 and PBP/PPARBP, a co-activator of the PPARα pathway, thereby regulating lipogenesis and cellular proliferation [120].

In the chicken liver, miR-22-3p contributes to lipid accumulation, unsaturated fatty acid biosynthesis, and PPAR-related signaling, with *ACSL5*, *ELOVL6*, and *PLIN2* identified as key hepatic targets [121,135] (Figure 5). Meanwhile, miR-146b-5p, located in an intergenic region on chromosome 6, shows elevated activity in the breast muscle of fast-growing broilers, linking it to rapid muscle growth and improved feed efficiency [116,136]. MiR-148a, abundantly expressed in hepatocytes, modulates multiple genes involved in lipid metabolism via WNT1 signaling [129,130,137]. Both miR-24-3p and miR-128-3p intersect pathways of muscle development and fat deposition by regulating the proliferation and inhibition of intramuscular preadipocytes [116,122].

Further examples include miR-34a-5p, which increases intracellular triglyceride and total cholesterol levels by suppressing translation of its hepatic target *ACSL1* [123], and miR-218-5p, which modulates ELOVL5 and FADS1, essential enzymes in the biosynthesis of long-chain polyunsaturated fatty acids [121,124] (Figure 5). Reduced expression of miR-101-2-5p elevates ELOVL5 levels in laying hens’ livers and suppresses ApoB, a critical component of lipoprotein assembly and secretion [124,138]. Additionally, miR-128-3p targets genes involved in glycerophospholipid metabolism and inhibits farnesyl diphosphate synthase (*FDPS*) [125].

Profiling of miRNA expression in abdominal fat from chicken lines selected for divergent fat content identified differentially expressed miRNAs, including miR-142-3p, miR-19a-3p, miR-19b-3p, miR-30d, miR-26a, miR-17-5p, miR-103-3p, miR-27b-3p, miR-92-3p, and miR-122-5p, whose targets, such as *ACSL1*, *FADS2*, and *ABCD3*, are central to lipid metabolism and abdominal fat deposition [129,139]. A broader set of miRNAs, including miR-429, miR-200b, miR-451, miR-142-5p, miR-200a, miR-218, miR-454, miR-30a-5p, miR-34, miR-199, miR-8, and miR-146b-5p, further regulate lipid metabolism and orchestrate adipocyte differentiation and proliferation [31,130].

Other miRNAs, such as miR-21, inhibit preadipocyte proliferation through downregulation of *KLF5* [129,130], while miR-223 and miR-18b-3p target *GPAM* and *ACOT13*, respectively, suppressing adipocyte differentiation [43,115]. Across developmental stages, specific miRNA-mRNA pairs, including miR-6701-3p-*PTEN*, miR-1563-*WWP1*, miR-6701-3p-*BMPR1B*, miR-29c-3p-*PIK3R1*, and miR-449c/d-5p-*TRAF6*, contribute to the dynamic modulation of fat deposition [131].

Together, these interconnected miRNAs form a complex regulatory network influencing adipogenesis, adipocyte proliferation and differentiation, hepatic lipid metabolism, intramuscular fat deposition, and abdominal fat accumulation in chickens.

### 4.2. Long Non-Coding RNA-Mediated Regulation of Adipogenesis

Long non-coding RNAs (lncRNAs) are single-stranded RNAs longer than 200 nucleotides, transcribed by RNA polymerases I, II, and III [140]. They may originate from various genomic regions, including enhancers, promoters, introns, or intergenic sequences [141,142,143]. Many lncRNAs are capped, spliced, and polyadenylated, exhibiting structural similarity to mRNAs, whereas others lack these features or derive from precursors containing introns or repetitive elements. Although generally less conserved evolutionarily, lncRNA genes share features with protein-coding genes, such as regulatory elements, multiple exons, chromatin signatures, and transcription factor regulation [144,145,146].

In chickens, lncRNAs have been shown to regulate adipogenesis and fat deposition through several mechanisms [147]. Nuclear lncRNAs contribute to transcriptional regulation, chromatin architecture, and nuclear organization, while cytoplasmic lncRNAs can act as competitive endogenous RNAs (ceRNAs) for microRNAs, scaffold proteins in signaling pathways, influence mitochondrial homeostasis, and modulate RNA splicing, stability, and localization [145,146].

Specific lncRNAs have been implicated in lipid accumulation in chickens. IMFNCR functions as a molecular sponge for miR-128-3p and miR-27b-3p, regulating PPARG expression and promoting differentiation of intramuscular preadipocytes [148]. MFNCR acts as a regulatory switch controlling muscle development and fat deposition by co-expressing with genes in PPAR signaling and facilitating preadipocyte differentiation via cis-activation of thioredoxin reductase 1 (*TXNRD1*) [149]. Differentially expressed lncRNA-46546 promotes triglyceride synthesis and lipid droplet accumulation in intramuscular preadipocytes by regulating its target gene *AGPAT2* (1-acylglycerol-3-phosphate O-acyltransferase 2) [150]. HLFF has been identified as a molecular sponge for miR-2188-3p through a ceRNA mechanism, modulating post-transcriptional activity of GATA-binding protein 6 (GATA6) and enhancing hepatic lipid synthesis [151]. LncRNA ZFP36L2-AS contributes to fat deposition by activating acetyl-CoA carboxylase alpha (ACACA) expression and inducing pyruvate carboxylase (PC) activity, which suppresses fatty acid oxidation [152].

### 4.3. Circular RNA-Mediated Regulation of Adipogenesis

Circular RNAs (circRNAs) are widely recognized as molecular sponges for miRNAs, thereby modulating the expression of their target genes [153]. Integrative analyses combining circRNA, miRNA, and mRNA expression profiles during chicken adipogenic differentiation have identified core competing endogenous RNA (ceRNA) networks. Functional enrichment analyses of these networks have underscored the involvement of key signaling pathways in fatty acid metabolism, notably PPAR and p53 pathways.

To elucidate non-coding RNA regulatory networks underlying fat deposition in chickens, whole transcriptome RNA sequencing was performed on abdominal fat, dorsal skin, and liver tissue from individuals exhibiting either low or high abdominal fat content [154,155]. ceRNA network analysis demonstrated that multiple metabolic processes are closely associated with fat accumulation in chickens, including fatty acid metabolism, monocarboxylic acid metabolism, carboxylic acid metabolism, glycerolipid metabolism, and PPAR signaling. Several pivotal genes, including *FADS2*, *HSD17B12*, *ELOVL5*, *AKR1E2*, *DGKQ*, *GPAM*, and *PLIN2*, were identified as central regulatory nodes and were shown to be targeted by miRNAs such as gga-miR-460b-5p, gga-miR-199-5p, gga-miR-7470-3p, gga-miR-6595-5p, gga-miR-101-2-5p. These miRNAs participate in competitive interactions with lncRNAs, including MSTRG.18043, MSTRG.7738, MSTRG.21310, MSTRG.19577, as well as with circRNAs, including novel_circ_PTPN2, novel_circ_CTNNA1, novel_circ_PTPRD [156].

### 4.4. Key Functional Circulating RNAs and Signaling Pathways

Key circRNAs have also been identified as important functional regulators of adipogenesis and lipid metabolism in chickens. For example, circDOCK7 enhances the expression of acyl-CoA synthetase long-chain family member 1 (ACSL1) through direct interaction with miR-301b-3p, thereby promoting the proliferation and adipogenic differentiation of chicken preadipocytes [157]. In addition, the chicken-specific circRNA circLCLAT1 acts as a miRNA sponge that prevents repression of essential adipogenic genes, including *RUNX1T1*, *FADS2*, *MYH9*, *IGF2BP3*, and *PDGFRA*, by miR-34a-5p, miR-30e-5p, miR-146b-5p, and miR-147. By releasing these genes from inhibitory miRNA control, circLCLAT1 promotes fat deposition. In essence, circLCLAT1 acts as a molecular sponge that removes inhibitory constraints imposed by miRNAs on adipogenic pathways, thereby representing an important regulator of obesity-related traits in poultry. Previous studies have shown that fibronectin type III domain containing 3B and 5 (FNDC3B, FNDC5) regulate browning and adipogenesis of white fat [158], while heparan sulfate proteoglycan 2 (HSPG2) modulates obesity and lipid deposition [159]. Other genes, including *RUNX1* translocation partner 1 (*RUNX1T1*), myosin heavy chain 9 (*MYH9*), fatty acid desaturase 2 (*FADS2*), and insulin-like growth factor 2 mRNA binding protein 1 (*IGF2BP1*), have also been associated with adipogenesis and fat accumulation [160,161,162]. CircLCLAT1 has been shown to influence adipogenic differentiation by regulating the expression of *RUNX1T1*, *FADS2*, *MYH9*, *IGF2BP3*, and *PDGFRA* (platelet-derived growth factor receptor alpha) through interactions with miR-34a-5p, miR-30e-5p, miR-146b-5p, and miR-147.

Overall, current knowledge on the role of circRNAs in chicken adipogenesis remains fragmented, and further in-depth studies are needed to elucidate the interacting circRNA–miRNA–mRNA networks that regulate metabolic pathways controlling fat deposition. One illustrative example is the IMFNCR/miR-128-3p/miR-27b-3p/PPARG ceRNA network, which plays a key role in the regulation of intramuscular fat deposition in chickens. This network operates according to the competing endogenous RNA (ceRNA) mechanism, in which a long non-coding RNA functions as a molecular sponge for specific miRNAs. The intramuscular fat–associated lncRNA IMFNCR is predominantly expressed in preadipocytes and promotes adipocyte differentiation in chickens by binding miR-128-3p and miR-27b-3p [148]. Furthermore, an additional regulatory axis involving LincRNA-MSTRG.673.2 and miR-128-3p has been identified, which similarly influences lipid metabolism in chickens [163].

## 5. Candidate Genes from Human Obesity and Comparative Genomics in Chickens

A comparative analysis of genes associated with human obesity and those present in chickens is provided in the Supplementary Table S3. Many genes linked to obesity in humans are also found in the chicken genome and contribute to excessive fat deposition in poultry (Supplementary Table S3).

The chicken genome was the first sequenced among non-mammalian vertebrates that is sufficiently close to mammals to allow meaningful comparative studies [164]. Sequencing the chicken genome enables reconstruction of ancestral mammalian genome architecture by using the chicken as an outgroup. Although the chicken genome is only about one-third the size of the human genome, it contains a comparable number of genes, approximately 20,000 to 23,000, which is similar to that of humans [165].

Chickens also exhibit several unique metabolic characteristics. They are naturally insulin-insensitive, which manifests as hyperglycemia, and they do not respond to high doses of exogenous insulin [166]. Additionally, chickens lack genomic loci for several key adipokines present in humans, including *LEP* [167], *SERPINE1*, *TNF*, *RETN*, *ITLN1*, and *ITLN2* [168]. This absence indicates that chickens rely on alternative genetic mechanisms to regulate processes such as food intake, appetite, and energy balance.

Core genes involved in the regulation of BW and lipid metabolism are conserved between the two species, yet species-specific genetic variants may differentially influence the development of obesity, reflecting adaptation to distinct ecological conditions and evolutionary histories [24]. The human obesity-related genes listed in Table S3 of Supplementary are orthologous to chicken genes (Supplementary Table S3). The product of the PPARG gene is a central regulator of adipogenesis and lipid metabolism in both chickens and humans. Despite this functional conservation, substantial structural and regulatory differences exist between the species. Structural variations include differences in gene size, organization, and genomic localization. Functional and tissue-specific expression patterns also diverge: in mammals, adipose tissue is the primary site of fat deposition, whereas in chickens, lipogenesis occurs predominantly in the liver (over 70%), with adipose tissue mainly serving as a storage depot. In humans, PPARG expression is highest in adipose tissue, while in chickens it is strongly expressed in adipose tissue, kidneys, and brain, and is also detected in immune organs. Skeletal muscle, in contrast, exhibits minimal PPARG expression in chickens [31]. Several genes involved in fat deposition regulation in mammals are absent or highly divergent in chickens. For example, the chicken LEP gene shares only 26–30% sequence similarity with its human ortholog. This suggests that chicken leptin acts predominantly via autocrine or paracrine mechanisms rather than as a circulating hormone, as in mammals. Experimental studies have shown that leptin does not significantly affect food intake in chickens, implying it does not play a major role in central appetite regulation [169]. The presence and function of other adipokines, including TNFα, resistin, and omentin, remain debated; however, visceral adipose tissue in broilers and laying hens consistently exhibits low adipokine expression [169]. Recent research indicates that birds utilize alternative regulatory cascades to maintain metabolic homeostasis. In chicken preadipocytes, one such pathway involves the activation and repression of KLF7 (GATA2, GATA3), representing a mechanism distinct from typical mammalian pathways. These factors modulate early adipocyte development by regulating KLF7 expression to balance proliferation and differentiation [170]. Adiponectin, visfatin, and chemerin have been cloned in chickens and act as key regulators of food intake and muscle growth [169]. Chickens also lack the insulin-dependent glucose transporter GLUT4, and their adipose tissue exhibits insulin resistance. Unlike mammals, insulin signaling in chickens is not significantly suppressed during fasting, suggesting alternative mechanisms of energy sensing. Non-coding RNAs, including circular RNA–microRNA–mRNA–ceRNA networks, may play important roles in adipocyte differentiation and tissue-specific fat deposition [171].

## 6. Molecular Targets for Genetic Selection and Chicken Breeding

Modern systems biology approaches allow the integration of comprehensive omics datasets to overcome the limitations of studies such as GWAS. This strategy not only provides insights into the molecular mechanisms underlying traits of practical importance, including abdominal fat percentage and feed conversion ratio, but also identifies molecular markers for chicken breeding [172].

Candidate genes associated with meat quality include *A-FABP*, *H-FABP2*, *PRKAB2*, and *LPL*. *A-FABP* is predominantly expressed in cardiac and skeletal muscle, where it binds fatty acids and plays a central role in fatty acid metabolism. Associations have been observed between single nucleotide polymorphisms (SNPs) in A-FABP and H-FABP and intramuscular fat content in chickens; specifically, the *A-FABP* BB and *H-FABP* CD genotypes are significantly associated with increased intramuscular fat, suggesting a potential interaction between these genes in regulating fat deposition. *PRKAB2*, a member of the protein kinase family, is involved in energy regulation, fatty acid oxidation, and glucose metabolism, and is recognized as a key determinant of meat quality in livestock [172]. Additionally, *FABP5*, *VNN1*, and *PKM2* have been validated as potential targets of miR-122 in the chicken liver, through which miR-122 modulates hepatic metabolism. The lncRNA IMFNCR acts as a molecular sponge for miR-128-3p and miR-27b-3p, thereby enhancing *PPARG* expression and directly promoting intramuscular adipocyte differentiation. This makes IMFNCR a primary target for increasing intramuscular fat, a critical factor in meat flavor and tenderness [173]. FASN is a key enzyme in de novo long-chain fatty acid synthesis and serves as a central node in the regulatory network of fat deposition. While its expression is often modulated by non-coding RNAs, such as miR-122, direct selection of *FASN* variants is used to control abdominal fat and improve meat quality. These molecular targets are increasingly employed in breeding programs to balance growth rate and meat quality, particularly to increase intramuscular fat while maintaining control over abdominal fat deposition.

## 7. Conclusions

The genetic selection of chickens with reduced abdominal fat deposition has emerged as a central objective in modern poultry production, driven by the goal of enhancing meat quality and overall productivity. Consequently, understanding the molecular mechanisms underlying abdominal fat accumulation is of critical importance. Over the past several decades, extensive research has been conducted at the cellular level, alongside comprehensive analyses of gene regulatory networks and expression profiles in abdominal adipose tissue. This body of work, summarized in the present review, provides valuable insights into the complex interplay of genes, transcription factors, and non-coding RNAs that govern fat deposition. Such knowledge not only deepens our understanding of adipogenesis in chickens but also lays the groundwork for the development of targeted breeding strategies aimed at optimizing growth efficiency while minimizing excessive fat accumulation, ultimately contributing to more sustainable and profitable poultry production.

## Figures and Tables

**Table 1:** Genes involved in adipogenesis and abdominal fat deposition in chickens.

Gene	Protein	Function
Lipogenesis pathway		
*FASN*	fatty acid synthase	de novo synthesis of fatty acids in the liver
*ACC*	acetyl-CoA carboxylase alpha	de novo fatty acid synthesis and desaturation
*SCD*	stearoyl-CoA desaturase	enzyme involved in fatty acid biosynthesis, primarily the synthesis of oleic acid
*CD36*	CD36 molecule fatty acid translocase (FAT)	transmembrane protein involved in fatty acid translocation, scavenging for oxidized fatty acids acting as a receptor for adhesion molecules
*APOA1*, *APOB*, *APOV1*	apolipoprotein AI apolipoprotein B apovitellenin	involved in fatty acid metabolism, including the metabolism of triglycerides
*FABP3*, *FABP4*, *FABP5*, *FABP7*	fatty acid-binding proteins	fatty acid handling and metabolic turnover
*GPAM*	mitochondrial glycerol-3-phosphate acyltransferase	catalyzes the first committed step of triglyceride synthesis by esterifying glycerol-3-phosphate.
*ELOVL1*, *ELOVL2*, *ELOVL5*, *ELOVL6*	ELOVL fatty acid elongase family	extend long-chain fatty acids and thereby influence abdominal fat deposition
Lipolysis pathway		
*LPL*	lipoprotein lipase	enzyme responsible for hydrolyzing triacylglycerols contained in circulating lipoproteins to produce free fatty acids
*CPT1*	carnitine palmitoyltransferase 1	enzyme that facilitates the mitochondrial import and subsequent β-oxidation of long-chain fatty acids
*ACADL*	acyl-CoA dehydrogenase, long-chain	initiates the mitochondrial β-oxidation of long-chain fatty acids
*MGLL*	monoglyceride lipase	hydrolyzing monoglycerides into free fatty acids and glycerol, promoting the mobilization of stored lipids
*APP*	amyloid beta precursor protein	transmembrane receptor that undergoes proteolytic processing to generate several bioactive peptides
*ACAT1*	acetyl-CoA acetyltransferase 1	participates in ketone-body metabolism and terpenoid biosynthesis
Transcription factors		
*SREBP1*	sterol regulatory element binding protein 1	regulator of lipid metabolism, directing the expression of genes involved in fatty-acid and cholesterol synthesis.
*PPARG*	peroxisome proliferator-activated receptor gamma	a principal regulator of adipocyte differentiation, lipid storage, and overall lipid homeostasis
*RXR*	retinoid X receptor family	ligand-activated transcription factors that regulate the biological effects of retinoids by their involvement in retinoic acid-mediated gene activation
*CEBPA*	CCAAT/enhancer binding protein alpha	regulates genes involved in lipid metabolism and glucose homeostasis
*CEBPZ*	CCAAT/enhancer-binding protein zeta	a transcriptional repressor, modulating key adipogenic regulators
*CEBPB*	CCAAT/enhancer-binding protein beta	facilitate adipocyte differentiation
*INSIG1*	insulin induced membrane protein	regulates cholesterol metabolism, lipogenesis, and glucose homeostasis, modulating the expression of lipogenesis enzymes
*FOXO1*	forkhead box O1	inhibiting PPARG transcriptional activity, controls genes involved in lipolysis and fatty-acid oxidation
*THRSP*	thyroid hormone responsive spot 14	regulates lipid metabolism
Mitochondrial and energy sensors		
*PRKAG2*	protein kinase AMP-activated non-catalytic subunit gamma 2	energy-sensing enzyme that monitors cellular energy status and functions by inactivating key enzymes involved in regulating de novo biosynthesis of fatty acid and cholesterol
*ATP5B*	ATP synthase, H+ transporting, mitochondrial F1 complex, beta polypeptide	enzyme catalyzed oxidation of metabolic substances like glucose, fatty acids and amino acids are transferred to electron carriers
*NDUFAB1*, *NDUFA9*	NADH:ubiquinone oxidoreductase subunit AB1 NADH:ubiquinone oxidoreductase subunit A9	play a critical role in mitochondrial function

**Table 2:** Overview of microRNAs modulating signaling pathways in adipogenesis.

Tissue	miRNA	Target Genes Count	Expression Change	Reference
Chick embryos (1–5 days old)	miR-363-3p	854	Increased	[117]
	miR-26a-5p	941		
	miR-10a-5p	228		
	miR-199-5p	473		
Chick embryos	miR-33-2-5p	524	Increased	[118]
	miR-33-3p	228		
Chick embryos	miR-122a	247	Increased	[119]
	miR-122b	227		
Chick embryos	miR-126-5p	874	Increased	[120]
	miR-17-5p	1077		
	miR-19b-3p	1314		
Liver	miR-22-3p	515	Increased	[121]
	miR-146b-5p	426		
Intramuscular preadipocytes	miR-24-3p	539	Decreased	[122]
	miR-128-3p	1142		
Liver	miR-34a-5p	706	Increased	[123]
Liver	miR-218-5p	928	Increased	[121]
Liver	miR-101-2-5p	529	Decreased	[124]
Liver	miR-148a-5p	341	Increased	[125]
	miR-21-5p	353		
	let-7F-5p	558		
	miR-26a-5	941		
	miR-30d	1207		
	miR-10a-5p	228		
Lung, ovary, duodenum, kidney, heart, liver, leg muscle, and breast muscle	miR-19a-3p	1314	Increased	[126]
Liver, adipose tissue, and breast muscle	miR-30a-5p	1207	Increased	[31]
Breast muscle tissue	miR-142-3p	331	Increased	[127]
Abdominal fat	miR-17-5p	1077	Increased	[31]
	miR-103-3p	1105		
	miR-92-3p	910		
Adipose tissue	miR-27b-3p	1295	Increased	[116]
Adipose tissue	miR-122-5p	247	Increased	[128]
Abdominal adipose tissue (fat-line and lean-line)	let-7a	1206	Increased in lean and fat broilers	[129]
	let-7j	882		
	let-7b	563		
	let-7c	1145		
	let-7k	1206		
Abdominal adipose tissue	miR-148a-3p	1200	Increased in lean broilers	[130]
Broiler preadipocytes (fat-line and lean-line)	miR-146c	251	Increased in lean broilers	[129]
	miR-222	502		
	miR-15a	1687		
	miR-206	675		
Abdominal adipose tissue (fat-line and lean-line)	miR-429	960	Increased in high-fat chickens	[11]
	miR-200b-3p	960		
	miR-451	28		
	miR-142-5p	592		
	miR-218-5p	928		
	miR-454-3p	1057		
Abdominal adipose tissue	miR-215-5p	138	Increased	[130]
Intramuscular adipose tissue	miR-223	468	Decreased	[43]
	miR-18b-3p	221		
Breast muscle tissue	miR-6701-3p	3992	Unknown	[131]
	miR-1563	398		

## Data Availability

No new data were created during preparation of this manuscript.

## Supplementary Materials

The following supporting information can be downloaded at: https://www.mdpi.com/article/10.3390/ani16020260/s1, Supplementary Table S1: Genome-wide loci associated with weight-gain traits in chickens; Supplementary Table S2: Filtered GWAS trait data in chickens; Supplementary Table S3: Key genes associated with body weight-related traits in chickens. Supplementary Figure S1: PRISMA flow diagram showing selection process of papers. Supplementary File S1: Script for data processing and visualization.

## Abbreviations

The following abbreviations are used in this manuscript:

AMPK	5′ AMP-Activated Protein Kinase
BW	Body Weight
CEBPA	CCAAT/Enhancer-Binding Protein Alpha
circRNA	Circular Ribonucleic Acid
CPT1	Carnitine Palmitoyltransferase 1
ELOVL	Very-Long-Chain Fatty Acid Elongase
FABP	Fatty Acid-Binding Protein
FASN	Fatty Acid Synthase
GPAM	Glycerol-3-Phosphate Acyltransferase
GWAS	Genome-Wide Association Studies
JNK	Jun N-Terminal Kinase
lncRNA	Long Non-Coding Ribonucleic Acid
MAPK	Mitogen-Activated Protein Kinase
miRNA	Micro Ribonucleic Acid
PPAR	Peroxisome Proliferator-Activated Receptor
QTLs	Quantitative Trait Loci
SHH	Sonic Hedgehog
SREBP	Sterol Regulatory Element Binding Protein
TGF1β	Transforming Growth Factor Beta 1
