# Supplementary figures and images for: Genomic Insights into Abdominal and Intramuscular Fat Deposition in Chickens and Their Implications for Productivity Traits: A Systematic Review
Source: Animals (Basel). 2026 Jan 15;16(2):260. doi: 10.3390/ani16020260 (PMC12837823; doi:10.3390/ani16020260)

Supplementary Figure S1. PRISMA flow diagram showing selection process of papers

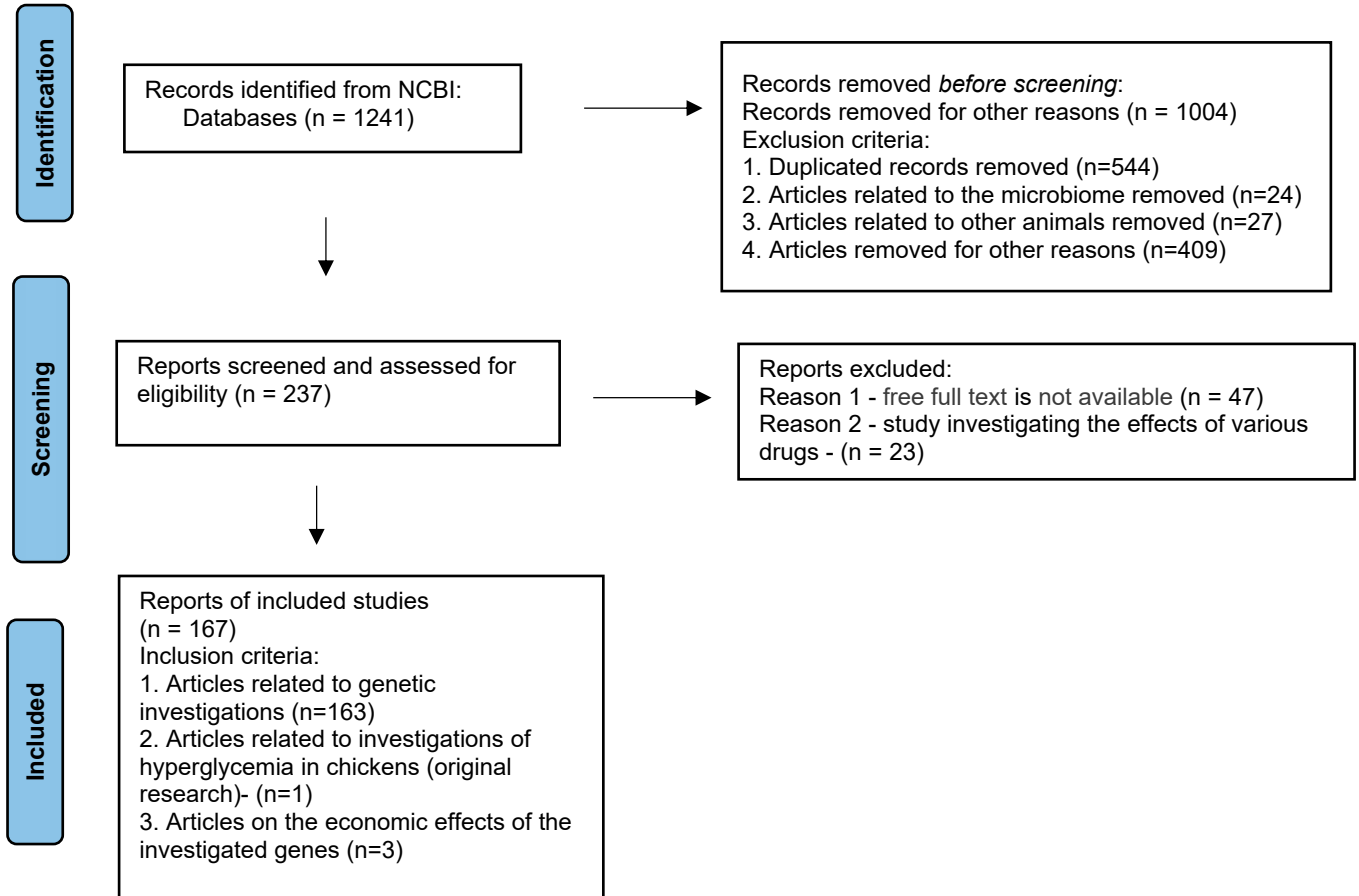

Supplement: Supplementary file 1 [file animals-16-00260-s001.zip › Supplementary_Fig-S1_22.12.2025.pdf]
